# Supplementary material for: Vaccinia Virus Arrests and Shifts the Cell Cycle
Source: Viruses. 2022 Feb 19;14(2):431. doi: 10.3390/v14020431 (PMC8874441; doi:10.3390/v14020431)
Supplement: Supplementary file 1 [file viruses-14-00431-s001.zip › viruses-1582038-supplementary.pdf]

viruses

Article

Supplementary: Vaccinia Virus Arrests and Shifts the Cell Cycle

Caroline K. Martin[1], Jerzy Samolej[2], Annabel T. Olson[3], Cosetta Bertoli[4], Matthew S. Wiebe[5], Robertus A. M. de Bruin[6], Jason Mercer[7*]

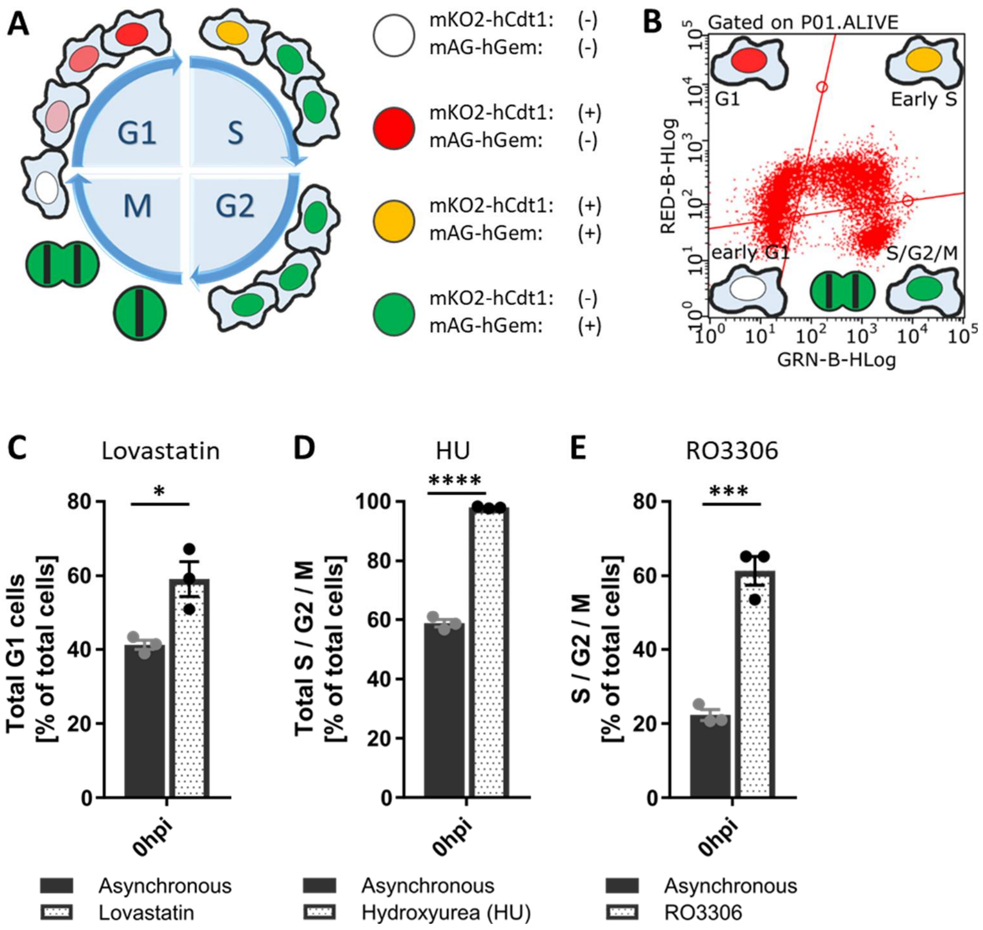

Figure S1. The HeLa FUCCI system for cell cycle analysis of synchronized and unsynchronized cells. [A] The HeLa FUCCI cell line stably expresses fluorescently tagged fragments of human Geminin (mAG-hGem(1-100), green) and Cdt1 (mKO2-hCdt1(30-120), red). Due to the antiphasic expression of the fragments, the relative abundance marks distinct phases of the cell cycle. The schematic shows cell cycle stage-dependent changes in fluorescence of the HeLa FUCCI cell line. Adapted from [50]. [B] Schematic flow cytometry fluorescence plot indicating the gating strategy used to group cells as either early G1 (no fluorescence), G1 (red), early S (green + red = yellow), and S/G2/M (green). [C-E] Synchronization effectiveness of Lovastatin, HU, and RO3306. [C] HeLa FUCCI cells were synchronized in G1 with Lovastatin (20 µM) for 24 h. Using flow cytometry, cells were classified as early G1, G1, early S, or S / G2 / M. Data represent the total G1 fraction (early G1 + G1) of either asynchronous, or Lovastatin treated cell populations. [D] HeLa FUCCI cells were synchronized in S phase with HU (2.5 mM) for 16 h and samples were analysed as in [C]. Data represent the total S / G2 / M fraction of either asynchronous, or HU treated cell populations. [D] HeLa FUCCI cells were synchronized in G2 phase with the CDK1 inhibitor RO3306 (10 µM) for 16 h and samples were analysed as in [C]. Data represent the S / G2 / M fraction (not including early S) of either asynchronous, or RO3306 treated cell populations. Experiments were conducted in biological triplicates and are displayed as mean ± S.E.M. Parametric, unpaired, two-tailed t-test for significance. ns. $p > 0.05$, * $p < 0.033$, ** $p < 0.0021$, *** $p < 0.0002$, **** $p < 0.0001$.

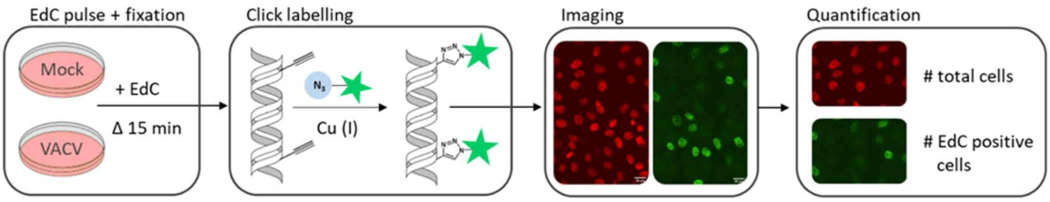

Figure S2. Experimental strategy to visualise cellular DNA synthesis in VACV infected cells. HeLa H2B-mCh cells were either mock infected or infected with VACV at MOI 8. 15 min before fixation, cells were pulse-labelled with the nucleotide analogue EdC (10 μM final). To detect nuclei with active DNA synthesis, incorporated EdC was covalently linked to AlexaFluor488 using the Click-iTTM Kit. Samples were imaged by confocal microscopy and the total amount of nuclei, marked by mCherry expression, as well as the number of EdC positive nuclei, marked by AF488 fluorescence, was determined.

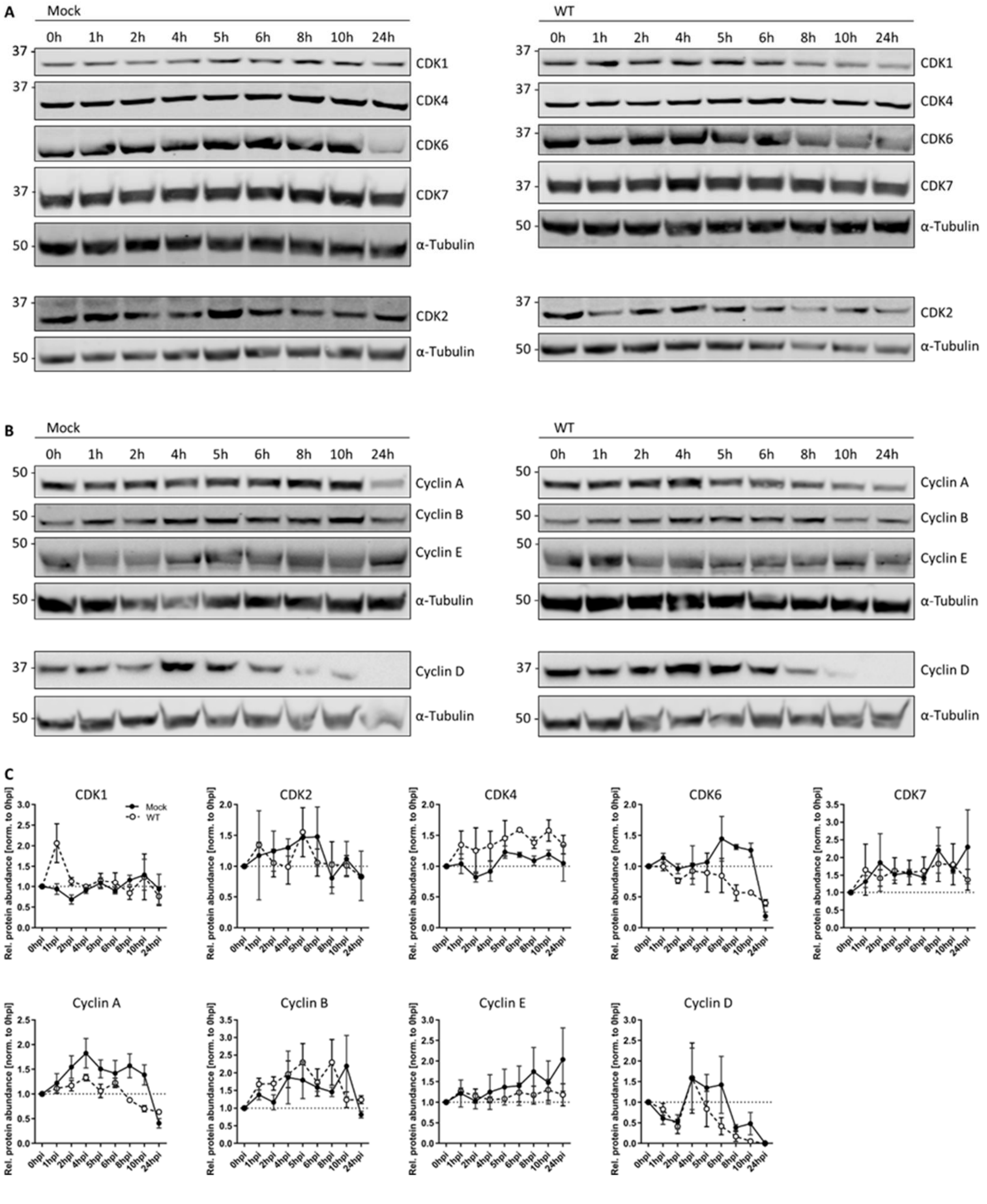

Figure S3. CDK and cyclin protein levels during VACV infection. [A-C] HeLa cells were either mock infected or infected with either WT VACV at MOI 5 and harvested between 0 h and 24 h. Whole cell lysates were resolved via SDS-PAGE and blotted for CDK 1, 2, 4, 6, 7, cyclin A, B, D, E, and α-Tubulin as loading control. [A-B] A representative blot of three biological replicates is shown. [C] Protein abundance was quantified and normalized to the loading control. Data represent biological triplicates, normalized to 0 h, and are displayed as mean ± S.E.M.

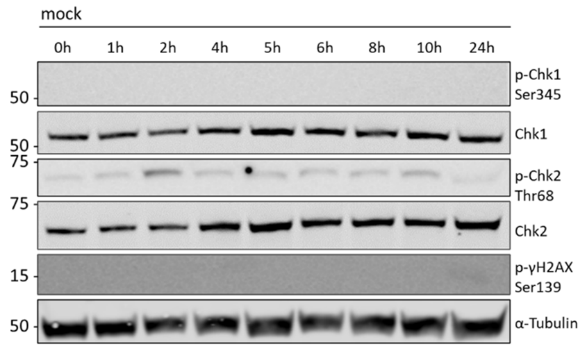

Figure S4. DDR activation level in mock infected HeLa cells. DDR activation was measured by phosphorylation of the DDR effector kinases Chk1 and Chk2, respectively. HeLa cells were mock infected and samples were harvested between 0 h and 24h. Whole cell lysates were resolved via SDS-PAGE and immunoblotted for activating phosphorylation of Chk1 (Ser345), Chk1, activating phosphorylation of Chk2 (Thr 68), phosphorylated γH2AX (Ser139) which marks cellular DNA damage, and α-Tubulin as loading control. A representative blot of biological triplicates is shown.

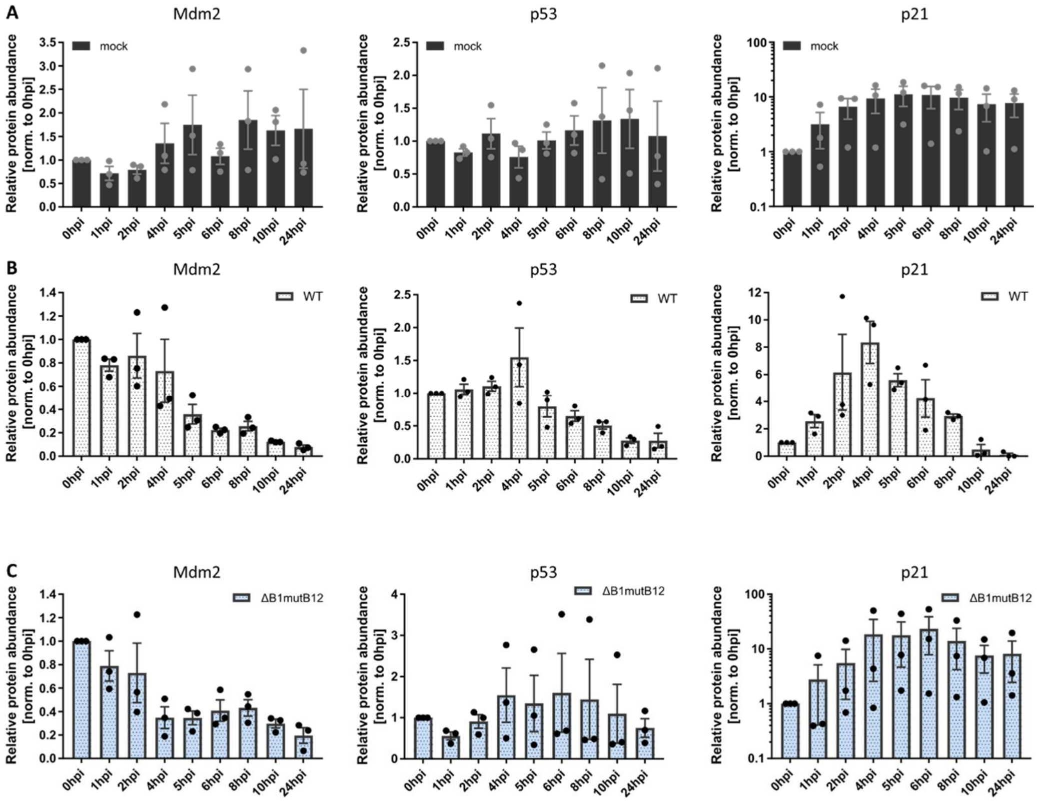

Figure S5. Quantification of Mdm2, p53, and p21 protein levels. [A-C] HeLa cells were either mock infected, or infected with either WT VACV, or VACV ΔB1mutB12 at MOI 5 and harvested between 0 h and 24 h. Whole cell lysates were resolved via SDS-PAGE and blotted for Mdm2, p53, p21, and α-Tubulin as loading control. Protein abundance was quantified and normalized to the loading control. Data represent biological triplicates, normalized to 0 h, and are displayed as mean ± S.E.M. [A] Mock infected control. [B] WT VACV. [C] VACV ΔB1mutB12 infection.

Table S1. Function of cell cycle inhibitors used in this study.

Cell Cycle Inhibitor	Description	Source / Reference
Mevinolin (Lovastatin)- 20 µM	Mevinolin acts as a potent, competitive inhibitor of hydroxymethylglutaryl-coenzyme A reductase (HMG-CoA). It is suggested to block cells in G1 by inhibiting the proteasome which causes accumulation.	LKT Labs, [40], [59]
DL-Mevalonic acid lactone (Mevanolate) - 6 mM	Addition of mevalonate to Mevinolin treated cells, releases the G1 block by re-stimulating proteasome activity.	Sigma-Aldrich, [59]
Hydroxyurea (HU) - 2.5 mM	Small molecule drug that inhibits the enzyme ribonucleotide reductase (RNR) which is required for synthesis of deoxyribonucleotides as precursors for DNA synthesis. Thereby, HU blocks (cellular) DNA synthesis and reversibly arrests cells in early S phase.	Sigma-Aldrich, [41], [42], [60], [61]
RO3306 - 10 µM	RO3306 is a small molecule inhibitor of CDK1 that reversibly arrests cells at the transition between G2/M.	Sigma-Aldrich, [64]

Table S2. Summary and description of VACV recombinants used in this study.

VACV Recombinant	Description	Reference
WR HA-D5	VACV WR encoding an endogenous HA-D5 fusion protein in the TK locus. No inhibition of the virus life cycle.	[44]
WR F10-SH EL EGFP	VACV WR encoding EGFP under a viral early late promoter in the TK locus, and a F10-Streptavidin-HA fusion protein in the endogenous F10L locus. No inhibition of the virus life cycle.	Mercer Lab unpublished
WR ΔB1mutB12	VACV WR B1R deletion virus which encodes a mCherry cassette instead of the B1R gene and contains an additional mutation in the B12R locus, which truncates the B12 pseudokinase protein. While a single B1 deletion virus is replication incompetent, the additional B12 truncation rescues viral replication.	[45]
WR Cts24	VACV WR encoding a thermolabile, endogenous D5 protein. Permissive temp: 31°C, non-permissive temp: 39.7°C. At non-permissive temperatures, virus fails to uncoate its genome, thus inhibiting subsequent virus life cycle steps such as genome replication and intermediate / late gene expression.	[44], [46]
v*ind*H1	VACV WR encoding the viral phosphatase H1 under an IPTG inducible LacZ promoter. Virus produced in the absence of IPTG lacks H1 in the virions and is strongly attenuated in viral early gene expression.	[38]
vL1Ri EL EGFP	VACV WR encoding L1 under an IPTG inducible LacZ promoter, and additionally encoding EGFP under a viral early late promoter. Virus produced in the absence of IPTG lacks L1 in the virions and is severely attenuated in fusion. Thus, infection is abortive at the entry step.	[47]
